# Evaluation of the Efficacy and Safety of Low‐Energy Versus High‐Energy Radiofrequency for the Treatment of Mild to Moderate Neck Wrinkles: A Split Neck Study

**DOI:** 10.1111/jocd.71096

**Published:** 2026-08-13

**Authors:** JingJing Tao, Zhenlai Zhu, Zhen Yi, Li Fan, Hongchun Peng, Bin Yang, Zhenfeng Liu

**Affiliations:** ^1^ Aesthetic Department Dermatology Hospital, Southern Medical University Guangzhou China; ^2^ State Key Laboratory of Oral & Maxillofacial Reconstruction and Regeneration, National Clinical Research Center for Oral Diseases, Shaanxi Key Laboratory of Stomatology, Department of Oral Medicine, School of Stomatology The Fourth Military Medical University Xi'an Shaanxi China

**Keywords:** anti‐aging, energy, neck skin laxity, plasma radiofrequency, wrinkles

## Abstract

**Background:**

Microplasma radiofrequency is frequently used for neck rejuvenation. However, the efficacy and safety of different energy protocols for neck treatment remain poorly understood.

**Objective:**

To explore the optimal energy during plasma radiofrequency for neck laxity treatment.

**Methods:**

Seventeen patients with mild‐to‐moderate neck wrinkles were enrolled in this split‐neck study. One side of each patient's neck was treated with low‐energy plasma radiofrequency in six sessions at two‐week intervals, while the contralateral side received high‐energy plasma radiofrequency in three sessions at four‐week intervals. Two dermatologists evaluated wrinkle improvement using Antera 3D imaging data collected before treatment and at 1, 3, and 6 months after the final session. Pain severity was assessed with a visual analog scale, and any post‐treatment adverse effects were recorded.

**Results:**

Both low‐ and high‐energy plasma radiofrequency treatments significantly improved neck wrinkles, with no significant difference in the degree of improvement between the two protocols. One month post‐treatment, the improvement rates from baseline were 18.5% (low‐energy) and 24.23% (high‐energy). At 3 months, the rates were 17.3% and 25.63%, respectively. Six months post‐treatment, the improvement rates were 24.37% for the low‐energy side and 15.45% for the high‐energy side. A significant difference in pain scores was observed between the two protocols (*p* < 0.05). Patients experienced mild pain on the low‐energy side and moderate pain on the high‐energy side. Erythema and edema were present on both sides immediately after treatment but subsided within 6 h. No adverse reactions, such as scabbing, post‐inflammatory hyperpigmentation, or scarring, were reported by any patients.

**Conclusion:**

Both low and high energy plasma radiofrequency can safely and effectively improve mild to moderate neck skin laxity. Low‐energy treatments have the advantage of causing less pain, while high‐energy treatments achieve the desired results with fewer sessions.

## 1 Introduction

Aging of the neck, a multifactorial process, is characterized by skin pigmentation, laxity, wrinkles, loss of jaw contour, and accumulation of fat under the chin. Factors such as genetics, vertical head movements, ultraviolet radiation, and pathologies involving neck tissue abnormalities contribute to the development of neck wrinkles. In aging skin, dermal collagen breaks down, the number of fibroblasts decreases, and collagen function and production are impaired, leading to laxity in the skin, which clinically manifests as wrinkles on the neck [1, 2]. Filler techniques, including autologous fat grafting, botulinum toxin injections, and hyaluronic acid fillers, are used to improve lateral neck wrinkles [3, 4, 5]. In addition, radiofrequency has emerged as a non‐surgical skin resurfacing treatment that uses radiofrequency energy to stimulate collagen production and improve skin texture and tone [6]. Plasma radiofrequency technology combines microplasma with monopolar radiofrequency to excite and dissociate nitrogen molecules in air into gaseous photons and electrons to form plasma. Placing a monopolar radiofrequency probe near skin tissue creates multiple controlled thermal damage channels, generating a thermal effect on deep collagen tissues, promoting collagen renewal and remodeling, ultimately improving skin texture and reducing wrinkles [7]. However, owing to the characteristics of the neck skin tissue structure, adverse reactions such as pain, secondary erythema, and hyperpigmentation may occur. Therefore, in this study, we aimed to determine the optimal energy for neck rejuvenation.

## 2 Materials and Methods

### 2.1 Study Design and Participants

Patients (*n* = 20, aged 25–50 years old) with mild‐to‐moderate neck skin laxity (neck wrinkle score ≥ 2 according to the Wrinkle Assessment Scale [3]) that presented to our hospital between August 2023 and June 2024 were enrolled into the study.

Patients who were pregnant or breastfeeding, had inflammatory skin lesions or open wounds in the treated area, had keloids or connective tissue disease, underwent laser or optical device treatments in the neck within the past 3 months, underwent radiofrequency, ultrasound, injections, or surgical treatments in the treated area within the past 6 months, or had serious systemic diseases or psychiatric‐related conditions were excluded from the study.

The patients' necks were divided into two sections bounded by the vertical midline and randomly assigned to receive either high‐energy or low‐energy treatment that was performed using a plasma radiofrequency therapy instrument (Fitton Medical Laser Ltd., Israel) and an ANTERA 3D detector (Miravex, Ireland). Random sequences are generated by the computer, and the treatment plans are matched according to these sequences. Subsequently, the assigned plans are printed out, placed in an opaque sealed envelope, and opened by the researchers based on the identification numbers of the subjects. Then, the treatment is carried out according to the assigned treatment plans. Throughout the entire trial, both the subjects and the treatment personnel are in a non‐blind state. Two dermatologists, who were not involved in patient recruitment or treatment, served as evaluating investigators and independently assessed the effectiveness of the treatment using ANTERA 3D data and clinical photographs of the subjects that were captured before treatment and at follow‐up.

### 2.2 Treatment Procedure

**FIGURE 1:**
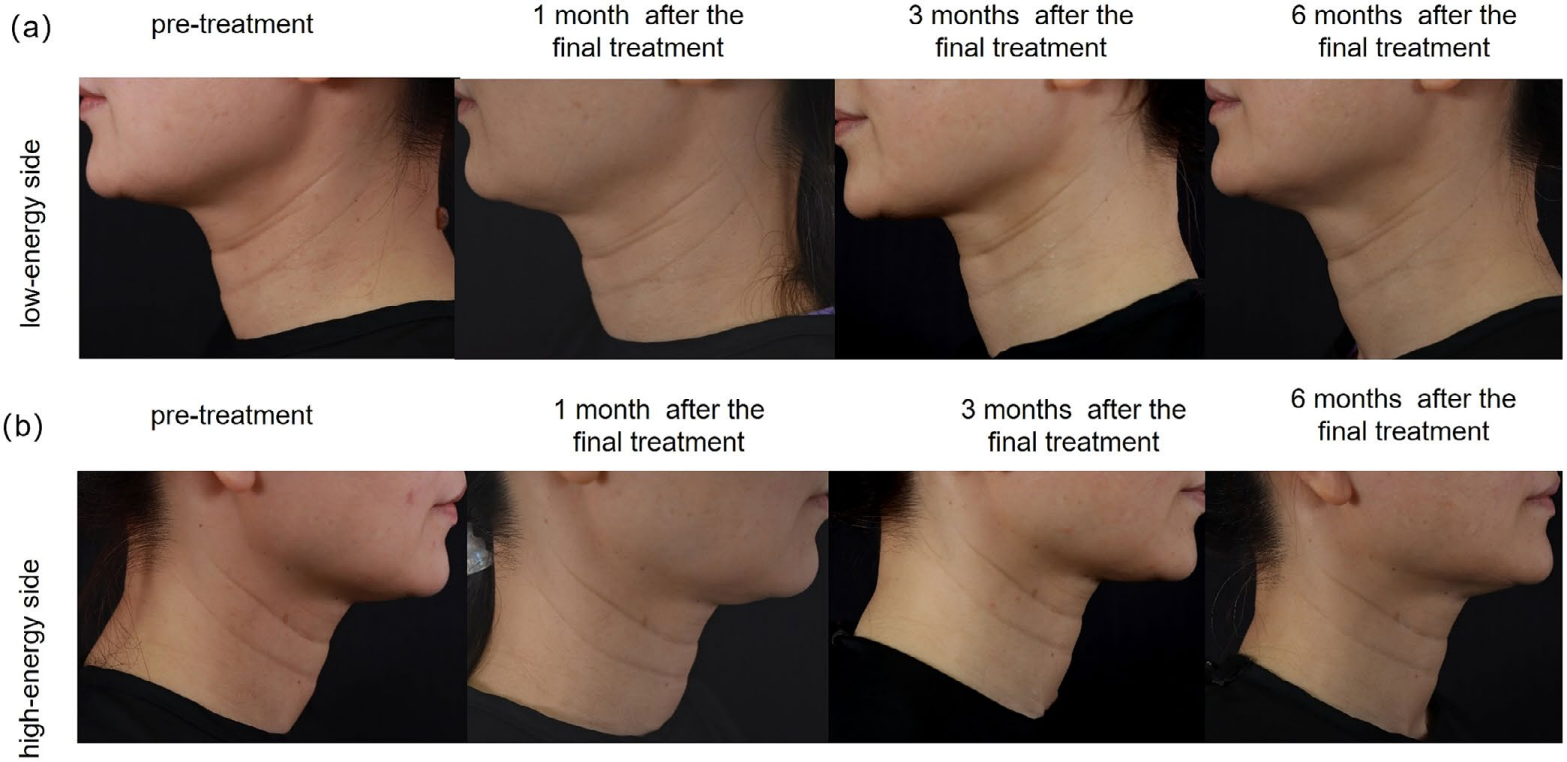
Clinical photograph of the neck at various time points during the follow‐up period. (a) low‐energy treatment and (b) high‐energy treatment side.

The participants were routinely cleaned and photographed before treatment. The treatment area was confirmed, and a compound lidocaine cream (Tongfang Pharmaceutical Group Co. Ltd.) was applied externally 40 min before treatment. During the treatment, the skin of the treatment area was cleaned, sterilized, and flattened, and the therapeutic handpiece was operated tightly against the skin. Both methods of treatment were conducted eight times with the R6 handpiece 1 mode, and the neck transverse wrinkles were superimposed three times with the R15 handpiece 2 mode, rolling at an average speed of 6 cm/s. The high‐energy treatment side was treated with 40 watts of therapeutic power once every 4 weeks for a total of three treatments, and the low‐energy treatment side was treated with 20 watts of therapeutic power once every 2 weeks for a total of six treatments. During the treatment period, patients completed a pain questionnaire for each treatment side, and the pain level was assessed using the visual analog scale (VAS). Other discomfort sensations during the treatment were recorded, followed by a telephonic interview to determine adverse reaction occurrences. Following treatment, follow‐up visits were performed at 1, 3, and 6 months, where ANTERA 3D tests were performed and clinical photographs were captured (Figure 1).

### 2.3 Efficacy Determination

The ANTERA 3D test was used to assess improvement of the neckline, and the degree of improvement was calculated as follows: (volume of neckline at baseline volume of neckline at follow‐up/volume of neckline at baseline) × 100. The overall improvement in neck skin was assessed using the Investigator‐Global Aesthetic Improvement Scale (IGAIS) [8]: grade 1, great improvement; grade 2, greater improvement; grade 3, improvement; grade 4, no change; and grade 5, deterioration.

### 2.4 Adverse Reaction Determination

The degree of pain was assessed using the VAS scale, with 0 indicating no pain, 1–3 indicating mild, 4–6 indicating moderate, 7–9 indicating severe, and 10 indicating severe unbearable pain. Adverse reactions that may have occurred during or after treatment were recorded.

### 2.5 Statistical Analysis

Descriptive statistical analysis (x̄±s) was used for the observed indicators and variables. The degree of improvement in the neckline and pain VAS scores was compared using the paired sample *t*‐test; the IGAIS score was compared using the non‐parametric rank sum test. Statistical analysis was performed using SPSS 23.0, and statistical significance was set at *p* < 0.05.

## 3 Results

### 3.1 Changes in the Degree of Neckline Improvement Before and After Treatment

**FIGURE 2:**
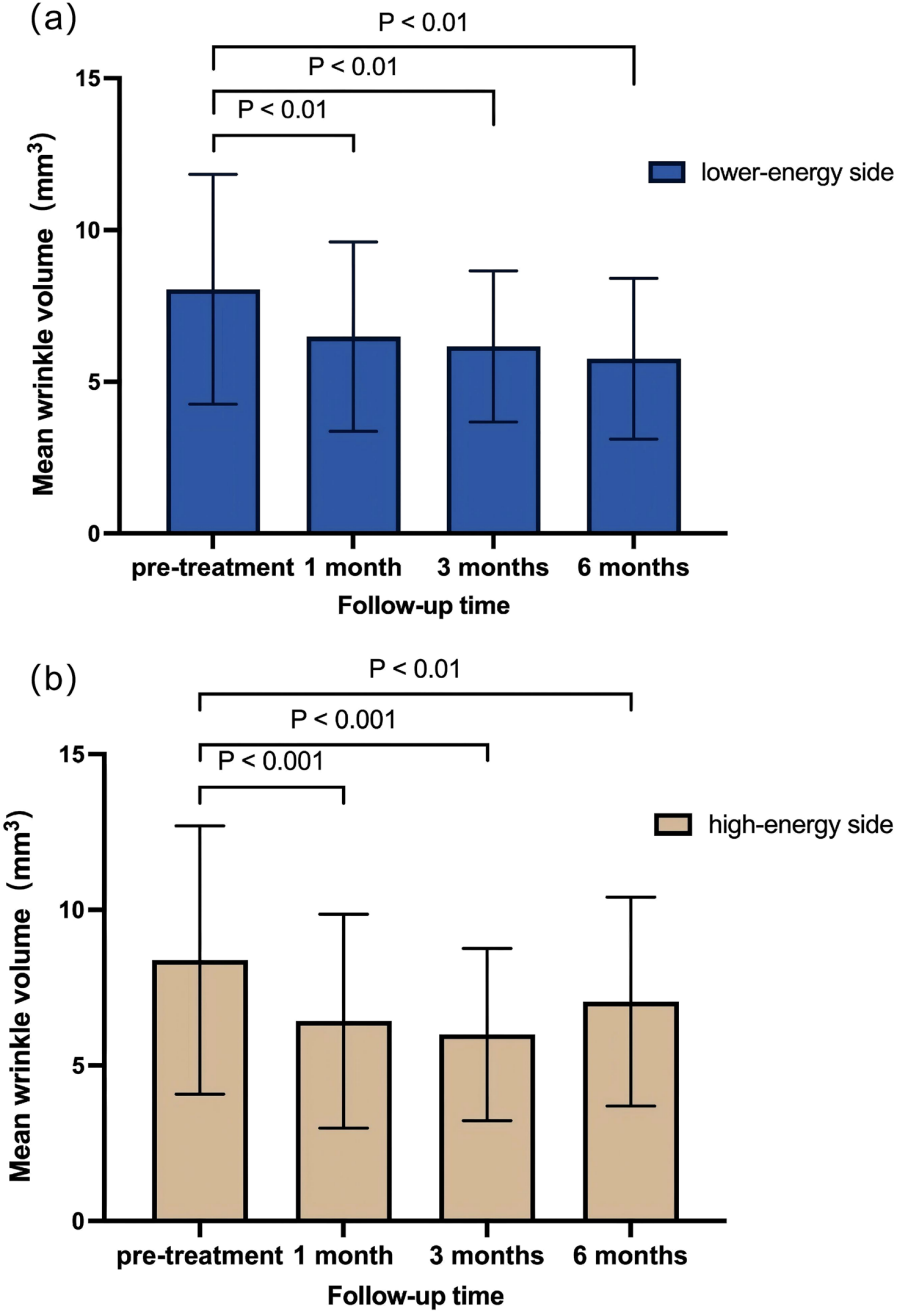
Comparison of mean changes in neck wrinkle volume between the two treatment groups during follow‐up. (a) low‐energy treatment and (b) high‐energy treatment side.

**FIGURE 3:**
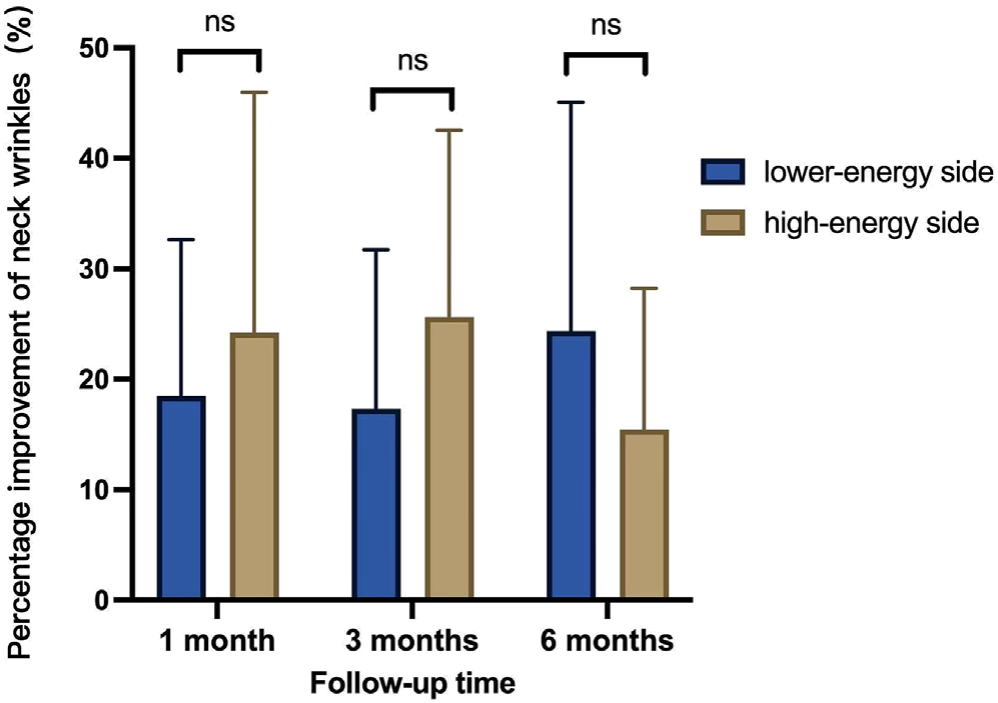
Comparison of neckline improvement rates between the low‐energy and high‐energy treatment groups during the follow‐up period. Neckline improvement rates = (wrinkle volume at baseline—wrinkle volume at follow‐up)/wrinkle volume at baseline × 100%. ns, Nonsense.

The ANTERA 3D results showed that, compared with pre‐treatment, the volume of wrinkles at the neckline decreased on both the low‐energy and high‐energy sides after treatment, and the neckline improved to a certain extent (Figure 2). The degree of improvement on the low‐energy side increased gradually with the duration of the visit, whereas that on the high‐energy side decreased at the 6th month. There was no significant difference in the degree of improvement between the two groups before and after treatment (Figure 3).

### 3.2 Changes in IGAIS Scores Before and After Treatment

**FIGURE 4:**
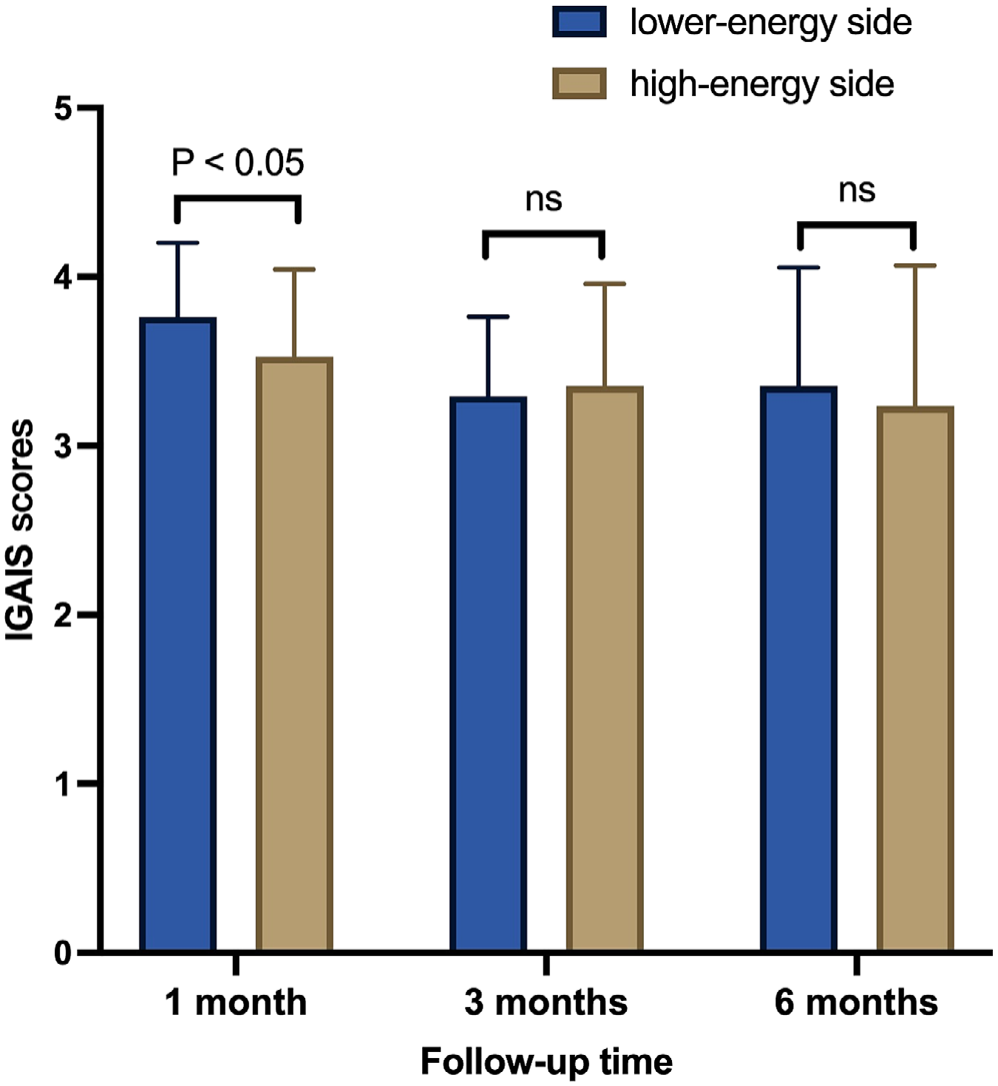
Comparison of Investigator Global Assessment of Improvement Scale (IGAIS) scores between the low‐energy and high‐energy treatment groups at various follow‐up time points.

In 17 patients, IGAIS scores for both the low‐ and high‐energy sides were lower after treatment. These improvements gradually became apparent over time and remained steady for 6 months. At 1 month, improvement on the high‐energy side was significantly better than on the low‐energy side, whereas this difference was not statistically significant at 3 and 6 months (Figure 4).

### 3.3 VAS Scores During Treatment

**FIGURE 5:**
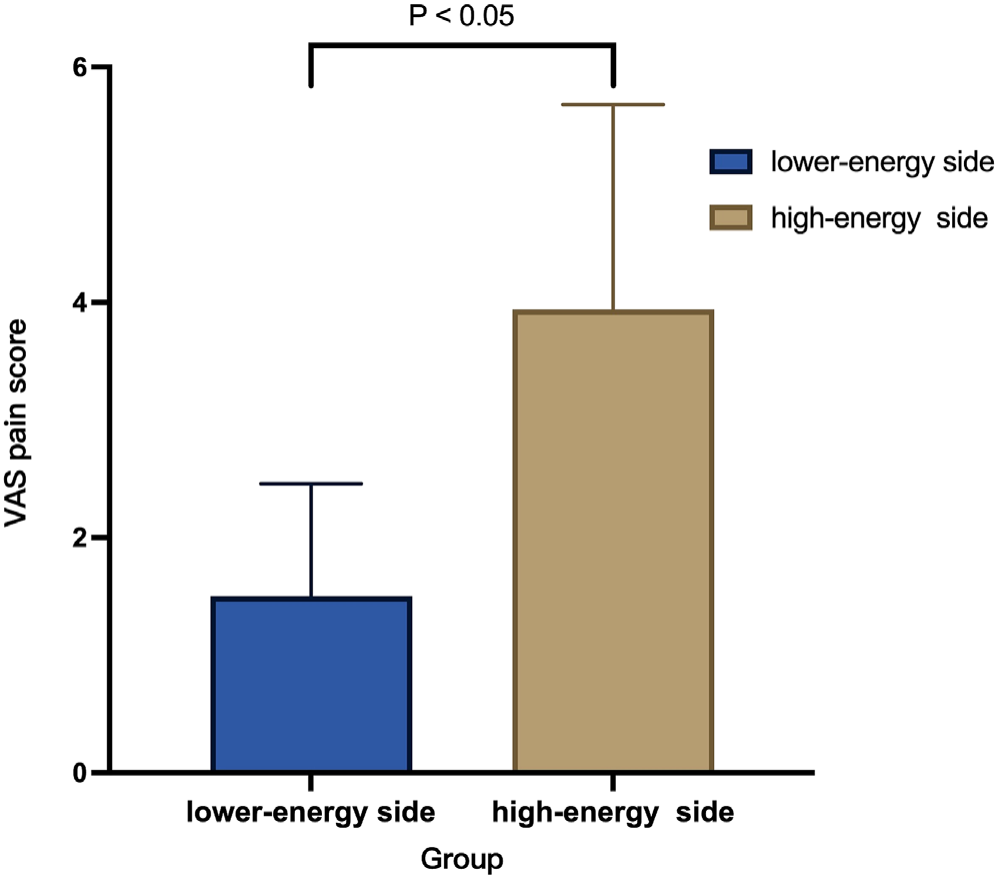
Comparison of mean Visual Analogue Scale (VAS) pain scores during treatment between low‐energy and high‐energy treatment groups.

Patients experienced mild pain (1.50 ± 0.96) on the low‐energy side and moderate pain (3.94 ± 1.74) on the high‐energy side, and there was a significant difference in pain scores between the two sides during treatment (*p* < 0.05) (Figure 5).

### 3.4 Postoperative Adverse Reactions

All patients experienced erythema and edema reactions on both sides immediately after treatment, which subsided within 6 h without treatment or intervention, and adverse reactions such as scabs, post‐inflammatory hyperpigmentation, and scarring were not observed.

## 4 Discussion

The neck has a complex meshwork that includes the papillary layer of the dermis, reticular layer, fibrous spacer network, subcutaneous fat, and the underlying fascia [9]. Together, these layers form the color and texture of neck [1, 10]. With aging, fat may accumulate in the neck and subchin region, leading to a loss of definition in the mandible. In addition, photoaging of the skin leads to epidermis thinning and a decrease in dermal collagen and elastin, resulting in an aged and flabby appearance [11]. Surgery is the primary treatment to remove excess skin and fat deposits from the neck [12]. Although effective, surgical treatment is associated with a long recovery time, trauma, and high cost [13, 14]. Thus, noninvasive or minimally invasive treatment modalities are needed [15]. Consequently, in recent years, photoelectric therapy has emerged as the primary method used for face and neck rejuvenation. Among these, micro‐plasma radiofrequency has become the main treatment option for neck rejuvenation owing to its advantages such as increased efficacy and fewer adverse reactions.

The therapeutic principle of micro‐plasma radiofrequency is the emission of monopolar radiofrequency to transform nitrogen into a plasma state, forming a micro‐exfoliation zone in the epidermis to the superficial dermis, initiating the mechanism of trauma repair in the organism, and simultaneously transferring heat to the deeper dermis, promoting the newborn and remodeling of collagen fibers, improving neck wrinkles, and tightening the skin [16]. Gentile et al. [17] reported that micro‐plasma radiofrequency is effective for face and neck rejuvenation with good patient tolerance and a short recovery time. Ruff et al. [18] reported a 74% patient satisfaction with helium plasma radiofrequency treatment.

The clinical impact of different treatment micro‐plasma radiofrequency protocols on the treatment of necklines has not been reported. Therefore, we aimed to determine the optimal micro‐plasma radiofrequency protocol for neck rejuvenation. Overall, we found that micro‐plasma radiofrequency treatment improved neck wrinkles; at 1 month, high‐energy treatment resulted in a greater improvement when compared to low‐energy treatment. Compared to pretreatment, neck wrinkles improved by 17.30% with low‐energy treatment and 25.63% with high‐energy treatment 3 months after treatment completion; however, the degree of improvement was not significantly different between the two treatment types. After 6 months of treatment, the degree of improvement increased with low‐energy treatment and decreased with high‐energy treatment, and the degree of improvement was not significantly different between the two treatment types. Consistent with previous findings [19, 20], our findings suggest that micro‐plasma radiofrequency produces both immediate and progressive thermal effects that persist for more than 3 months, while collagen regeneration persists for up to 6 months. This therapeutic model has direct clinical significance regarding the selection of microparticle radiofrequency energy: for patients who wish to see significant therapeutic effects in a shorter period of time, high‐energy treatment can be chosen; while low‐energy treatment achieves longer‐lasting effects through the gradual formation and remodeling of collagen, and has better tolerance.

In the present study, all patients experienced adverse reactions such as erythema and edema after treatment because micro‐plasma radiofrequency causes microexfoliation damage to the treatment area. These adverse effects were experienced for a longer time with high‐energy treatment but subsided within 6 h without causing severe damage or delayed healing. None of the patients experienced serious adverse effects such as crusting, post‐inflammatory hyperpigmentation, scarring, or infection. In addition, the pain was more intense on the high‐energy side than on the low‐energy side, with most subjects experiencing moderate pain during high‐energy treatment. Three subjects opted out of the treatment course because they could not tolerate the pain. This study is associated with some limitations, including the small sample size, single‐center design, and skewed population (all patients were female). Therefore, large‐sample multicenter clinical studies are needed to validate the study findings.

Nonetheless, in summary, both low and high micro‐plasma radiofrequencies effectively improved neck wrinkles without causing serious adverse effects. The efficacy of low and high energy treatment strategies was comparable. However, the low energy treatment was associated with mild pain during treatment, better comfort, shorter postoperative erythema, and a faster recovery period. Therefore, treatment methods can be rationally selected according to the patients' needs and skin conditions.

## Author Contributions

Research plan design: Z.L, B.Y., J.T. and Z.Z. Analyzed and interpreted the data: J.T. and Z.Z. Wrote the article: J.T. and Z.Z. Data collection: Z.Y. Therapist: L.F. and H.P., J.T. and Z.Z. contributed equally to this study. All authors read and approved the final manuscript.

## Funding

No funding was received for this research.

## Ethics Statement

This clinical study was approved by the Ethics Committee of the Dermatology Hospital of Southern Medical University (No. 2022111), and all subjects participated voluntarily and provided informed consent.

## Consent

All patients included in this study provided written informed consent for the publication of their clinical photographs. The images are used solely for scientific presentation and illustration of treatment outcomes, with no identifying information disclosed to protect patient privacy and confidentiality.

## Conflicts of Interest

The authors declare no conflicts of interest.

## Data Availability

The data that support the findings of this study are available from the corresponding author upon reasonable request.
